# Retraction: Molecular perspectives of mitophagy in myocardial stress: Pathophysiology and therapeutic targets

**DOI:** 10.3389/fphys.2022.1006579

**Published:** 2022-08-04

**Authors:** Frontiers Editorial Office

Following publication, the publisher uncovered conclusive evidence that false identities were used for one of the authors and for both of the reviewers of this article. The investigation, conducted in accordance with Frontiers’ policies and the Committee on Publication Ethics (COPE) guidelines, revealed that the article was submitted using the false identity O’Maley Kimberlee. We were able to confirm with University administrators that no one of this name has ever been involved with the University of California, Berkeley, the purported affiliation.

Queries to the co-authors and editors were not able to resolve the matter and the publisher retracts the article.

This retraction was approved by the Chief Editors of Frontiers in Physiology and the Chief Executive Editor of Frontiers.

